# The use of checklists in the intensive care unit: a scoping review

**DOI:** 10.1186/s13054-023-04758-2

**Published:** 2023-11-30

**Authors:** Ethan J. Erikson, Daniel A. Edelman, Fiona M. Brewster, Stuart D. Marshall, Maryann C. Turner, Vineet V. Sarode, David J. Brewster

**Affiliations:** 1 Intensive Care Unit, Cabrini Hospital, Malvern, Melbourne, Australia; 2 Department of Critical Care, Alfred Health, Melbourne, Australia; 3 Department of Anaesthesia, The Royal Women’s Hospital, Parkville, Melbourne, Australia; 4 Department of Critical Care, University of Melbourne, Melbourne, Australia; 5 Department of Anaesthesia, Peninsula Health, Melbourne, Australia; 6 Department of Anaesthesia, The Royal Children’s Hospital, Melbourne, Australia; 7 Central Clinical School, Faculty of Medicine, Nursing and Health Sciences, Monash University, Melbourne, Australia

**Keywords:** Checklist, Intensive care, Scoping review, Rounding, Delirium screening, Handover, Infection prevention, Airway management

## Abstract

**Background:**

Despite the extensive volume of research published on checklists in the intensive care unit (ICU), no review has been published on the broader role of checklists within the intensive care unit, their implementation and validation, and the recommended clinical context for their use. Accordingly, a scoping review was necessary to map the current literature and to guide future research on intensive care checklists. This review focuses on what checklists are currently used, how they are used, process of checklist development and implementation, and outcomes associated with checklist use.

**Methods:**

A systematic search of MEDLINE (Ovid), Embase, Scopus, and Google Scholar databases was conducted, followed by a grey literature search. The abstracts of the identified studies were screened. Full texts of relevant articles were reviewed, and the references of included studies were subsequently screened for additional relevant articles. Details of the study characteristics, study design, checklist intervention, and outcomes were extracted.

**Results:**

Our search yielded 2046 studies, of which 167 were selected for further analysis. Checklists identified in these studies were categorised into the following types: rounding checklists; delirium screening checklists; transfer and handover checklists; central line-associated bloodstream infection (CLABSI) prevention checklists; airway management checklists; and other. Of 72 significant clinical outcomes reported, 65 were positive, five were negative, and two were mixed. Of 122 significant process of care outcomes reported, 114 were positive and eight were negative.

**Conclusions:**

Checklists are commonly used in the intensive care unit and appear in many clinical guidelines. Delirium screening checklists and rounding checklists are well implemented and validated in the literature. Clinical and process of care outcomes associated with checklist use are predominantly positive. Future research on checklists in the intensive care unit should focus on establishing clinical guidelines for checklist types and processes for ongoing modification and improvements using post-intervention data.

**Supplementary Information:**

The online version contains supplementary material available at 10.1186/s13054-023-04758-2.

## Background

Checklists are a type of cognitive aid used to ensure that all components of a particular task are completed to promote adherence to best practices and prevent errors of omission [1, 2]. Checklists are well known to improve leadership and followership, team performance, and patient outcomes within wider healthcare [3, 4]. The ICU is a complex clinical environment comparable to the aviation industry which has a long history of checklist use [5, 6]. Indeed, the use of checklists in aviation has been shown to improve performance and safety over many decades and, as a result, checklists have been adopted within the ICU environment [7]. Research over the past two decades also has supported the efficacy of some specific checklists within the ICU environment [8–11].

Modern intensive care requires health care workers to work from a multiplicity of checklists that are designed to aid the completion of a range of complicated clinical tasks. Even within a particular checklist, there is a diversity of checklist items, implementation methods, and outcomes measured. Consequently, deciding what specific checklists should be used and how they should be implemented within an ICU becomes understandably complex. There are currently no published reviews examining the broader role of checklists within the ICU, their implementation and validation, and the recommended clinical context for their use.

This scoping review aims to present the principal types of checklists used in the ICU, any implementation and validation data as well as outcomes associated with their use. Furthermore, this review aims to determine the direction of further research into checklists within the ICU. The specific research questions (RQs) that this paper aims to address are as follows:

1. What checklists are used in the ICU?
2. How are checklists utilised in the ICU?
3. Do checklists influence clinical or process of care outcomes in the ICU?
4. What is the evidence for the implementation and validity of checklists in the ICU?
5. What further research is required for checklists in the ICU?

## Methods

### Search strategy

This scoping review was conducted in accordance with the Preferred Reporting Items for Systematic Reviews and Meta-Analyses Extension for Scoping Reviews (PRISMA-ScR) guidelines [12]. A completed PRISMA-ScR checklist is included in Additional file 1: Appendix S1. A protocol was not registered. A systematic search of MEDLINE (Ovid), EMBASE, Scopus, and Google Scholar was in May 2023. The search strategy employed a combination of keywords and Medical Subject Headings (MeSH) terms specific to checklists, intensive care units, and their respective synonyms and hyponyms (Additional file 1: Methods). The database search was supplemented by articles identified through reference list screening and a manual grey literature search on Google and Google Scholar. In the grey literature search, we also searched for checklists endorsed by major intensive care societies (Additional file 1: Appendix S2).

### Inclusion and exclusion criteria

For the purposes of this study, a checklist was defined as a structured list of actions presented in a physical or digital format for healthcare workers to follow [13]. The inclusion criteria were as follows: (1) examines the use of checklists; (2) is set in an ICU; (3) is a research study (no methodological restrictions), review article, meta-analysis, or guideline; (4) contains an abstract; (5) published in or since 2012; and (6) written in English.

A study was excluded if: (1) a checklist was not recommended, studied, or implemented; (2) the checklist mentioned was not designed for clinical use; or (3) it was not set in a clinical setting (e.g. simulation studies). Abstracts in conference presentations were excluded.

### Study selection

The titles and abstracts of eligible studies were screened by one reviewer (EE) using the inclusion and exclusion criteria. A 10% sample was screened by a second reviewer (DB) to assess concordance between the two reviewers for quality assurance. The studies selected for full-text review were reviewed by one reviewer (EE) using the inclusion and exclusion criteria and discussed with a second reviewer (DB). The references of studies that passed full-text review were screened for relevant references and additional relevant papers were included.

### Data synthesis

Data from the included articles were extracted, charted, and mapped. A data extraction form was developed by EE and DB to extract the year and country of publication, study design, intervention, and results from all included articles. A second data extraction form was developed through iterative discussion and feedback from all authors to collect more information on the articles that were grouped into one of the five categories of checklists. This second form was used to chart checklist characteristics, which ICU providers use them, adherence, development, use of study data to improve the checklist, details of implementation, and significant outcomes.

Both data extraction forms were successfully pilot tested on a sample of 15 articles before use. In order to plan for the heterogeneity of study designs and interventions, “NA” was designated when a section of a form was not applicable to a particular article. For instance, systematic reviews which examined multiple unique checklists from different studies were not applicable to sections such as checklist length or development. This was done with the intent to not duplicate results in case a systematic review covered an article already included in this review. We decided to include literature reviews in this review for completeness; however, they were not applicable to most sections of the extraction forms except for year, country, study design due to their nature. Descriptive statistics were used to tabulate and graph the data collected in the data extraction forms.

## Results

**Fig. 1:**
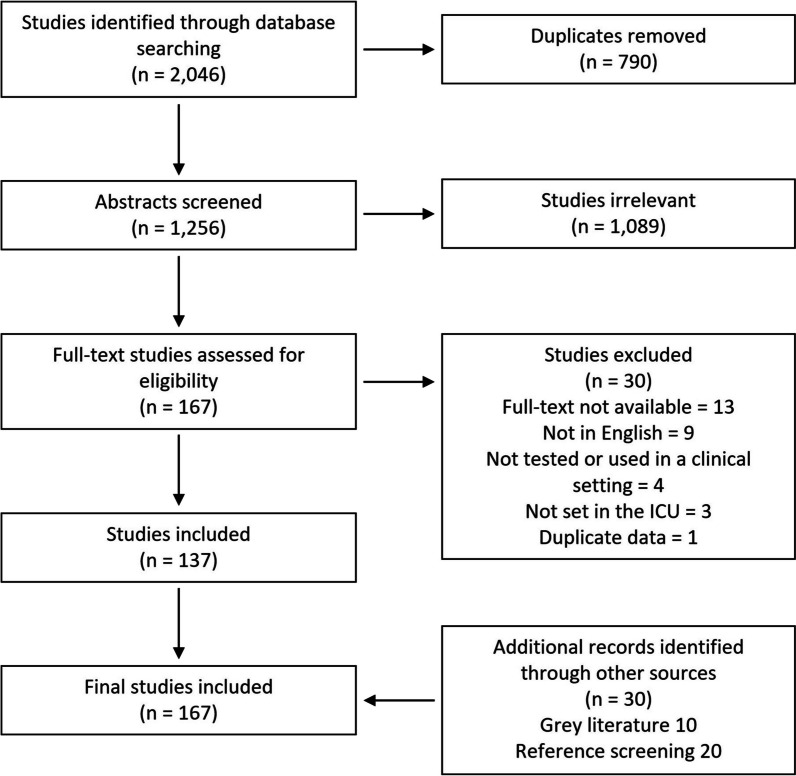
PRISMA flow diagram

Our search identified 2046 abstracts through database searches. A total of 790 duplicates were initially removed and a further 1089 articles were excluded during title and abstract screening. Of the remaining 167 full-text articles assessed for eligibility, 137 articles were included in addition to 30 articles identified through reference screening and a grey literature search (Fig. 1). There was 100% agreement between reviewers when the second reviewer screened a 10% sample. The titles of the 167 included articles are provided in Additional file 1: Appendix S3.

### Origin of research

The distribution of studies produced by each continent is shown in Additional file 1: Figure S1. Notably, 45% (75/167) of studies were produced by the USA. Within any geographical region, there were no healthcare organisations stood out in their use of these checklists. The number of articles published on ICU checklists has exhibited an upward trend for the past decade and peaked at 22 studies in 2018 (Additional file 1: Figure S2).

### Types of checklists identified

Checklists identified within the included articles were broadly used to guide staff performance and clinical procedures (e.g. rounding behaviour or airway management), or to facilitate patient assessment (e.g. delirium screening tools). Rounding checklists are performed on ward rounds and typically focus on the routine daily care plans for patients within ICU. Checklists in delirium screening tools are entirely composed of the intensive care delirium screening checklist (ICDSC). Transfer or handover checklists facilitate the safe transfer of patient care from the ICU to another clinical environment or the handover of care between shifts. CLABSI prevention checklists focus on reducing the rate of CLABSIs and/or catheter-related bloodstream infections (CRBSIs) and are predominantly centred around the processes of central line placement, maintenance, and necessity. Airway management checklists are primarily concerned with the intubation and extubation of ICU patients.

**Fig. 2:**
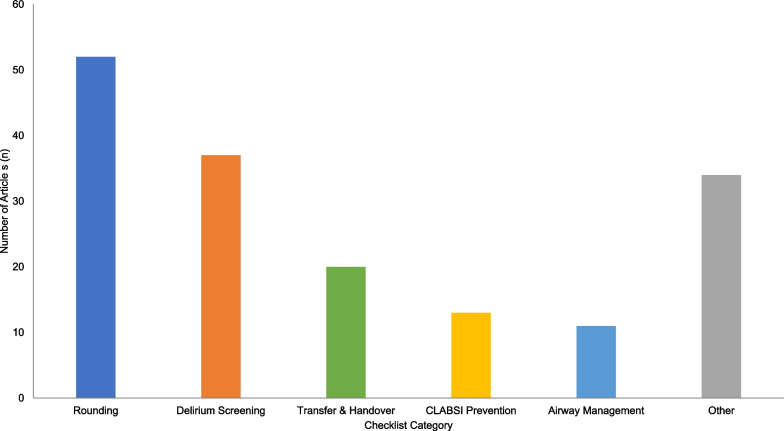
Articles by checklist category (*n*)

Figure 2 demonstrates the proportion of checklists described within the 167 articles by clinical category, with rounding and delirium screening checklists being the most prevalent.

A total of 133 articles examined checklists specific to rounding, delirium screening, transfer/handover, CLABSI prevention and airway management. The sample size of these 133 articles is shown in Additional file 1: Appendix S4. These papers were further analysed for checklist content, media, implementation, development and associated clinical outcomes. The papers were divided into the categories of “primary articles” (*n* = 123) and “guidelines” (*n* = 10) for this analysis.

### Checklist content

Sixty-nine unique checklists were identified within the 123 primary articles. Checklists were most commonly comprised of six to 20 discrete checklist items (median: 17, range: 3–56) (Additional file 1: Figure S3). The most frequently reported checklist was the standardised ICDSC, a delirium screening checklist comprised of 8 items, which appeared in 32 analysed articles. Four unique checklists were identified within the ten included guidelines. These checklists comprised an average of 41 discrete checklist items (median: 41.5, range: 8–73).

### Use of checklists identified

#### Medium

**Fig. 3:**
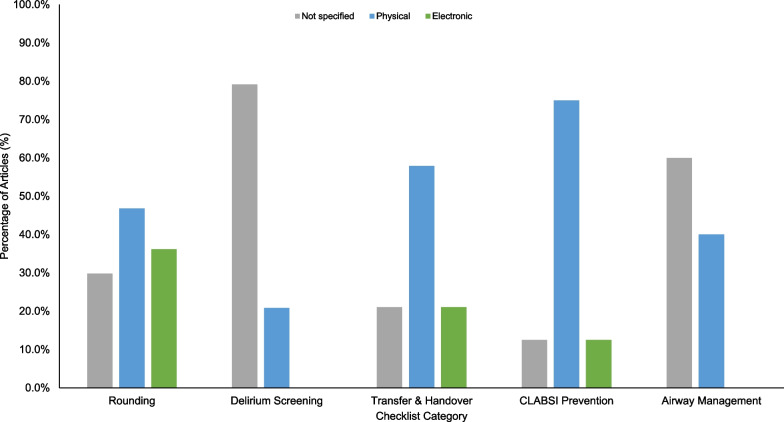
Percentage of articles that used physical and/or electronic checklists

Physical checklists were the most prevalent across all checklist types (Fig. 3). However, electronic checklists have been increasingly studied over the past decade (Additional file 1: Figure S4) while research on physical checklists has been decreasing (Additional file 1: Figure S5).

Five dynamic checklists integrated into the electronic health record (EHR) were identified, generally as part of a clinical decision support tool [14–18]. Four of the checklists were rounding checklists and one was a CLABSI prevention checklist. These checklists present items relevant to a particular patient, autocomplete elements of the checklist based on existing electronic documentation or EHR data, or provide relevant EHR information alongside checklist items to aid clinical decision-making.

#### Who uses checklists in the ICU?

**Fig. 4:**
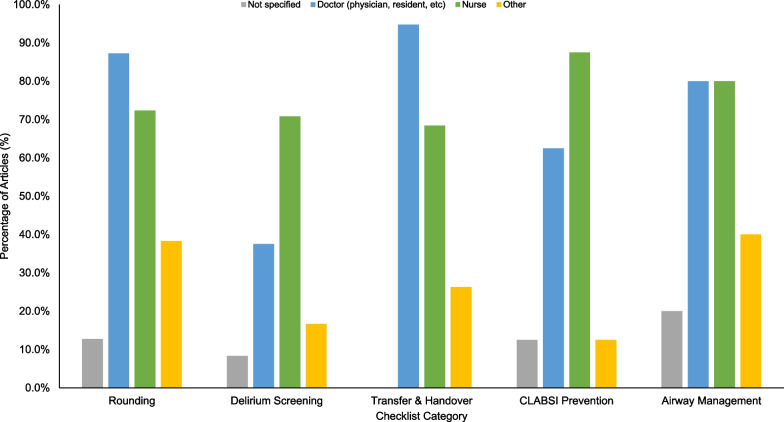
Checklist usage by healthcare worker

Doctors were most involved with the use of rounding checklists and transfer/handover checklists while nurses were most involved in completing the delirium screening and CLABSI prevention checklists (Fig. 4). Airway management checklists were reported as being used equally between doctors and nurses. Additionally, other healthcare providers such as pharmacists and respiratory therapists were involved in the use of all checklist types to varying degrees.

#### Adherence

43% (34/79) of studies reported adherence rates. Adherence was defined as the completion or use of a checklist. The median adherence and duration of adherence measurement for each checklist is presented in Table 1.

**Table 1:** Checklist compliance

	Rounding	Transfer & handover	CLABSI prevention	Airway management
Compliance, median (%)	90.6	84.7	81.5	73.0
Duration of measurement, median (months)	9	5	10.5	10

While some articles commented on a culture of quality and safety in the ICU, few commented on the possibility of a pre-existing safety culture contributing to checklist adherence. However, these articles frequently reported using initiatives to foster a culture of safety alongside implementation of the checklist intervention. Furthermore, many found that checklist itself improved safety culture.

### Outcomes of checklist use

The statistically significant clinical and process of care outcomes reported by the 123 primary articles were categorised into positive, negative, and mixed by the first author (EE) and verified by the second author (DB) (Table 2). Positive and negative outcomes were defined as outcomes that significantly improved or deteriorated after checklist implementation, respectively. Mixed outcomes refer to outcomes that exhibited both a significant improvement and deterioration after checklist implementation (e.g. an outcome improved in hospital A but worsened in hospital B). Most articles reported significant positive outcomes across all checklist types while few reported significant negative or mixed outcomes.

**Table 2:** Articles that reported a statistically significant outcome for each checklist type

Significant outcomes	Rounding	Delirium screening*	Transfer & handover	CLABSI prevention	Airway management
Positive/Improve, *n* (%)	37 (71.2)	26 (81.3)	15 (75.0)	7 (63.6%)	4 (50.0)
Negative/Worsen, *n* (%)	6 (11.5)	6 (18.8)	0 (0.0)	1 (9.1)	1 (12.5)
Mixed, *n* (%)	2 (3.8)	6 (18.8)	0 (0.0)	0 (0.0)	0 (0.0)

^*^Outcomes of delirium screening checklist articles were reported in Table 2 regardless of their significance to best reflect their findings. Few delirium screening checklist articles reported significant outcomes with 34.4% (*n* = 11) reporting significant positive outcomes, 12.5% (*n* = 4) reporting significant negative outcomes, and 6.3% (*n* = 2) reporting significant mixed outcomes

#### Outcomes from delirium screening checklists

The purposes of delirium screening checklist studies were predominantly related to validity or reliability, which naturally yielded few significant outcomes. Positive outcomes typically referred to findings of a delirium screening checklist being valid and/or reliable. Negative outcomes refer to outcomes such as predictive validity or reliability of the delirium screening checklist being negatively influenced by patient characteristics such as sedation or neurological deficits. Mixed outcomes were classified as a positive and negative finding occurring at the same time (e.g. the ICDSC displayed a high sensitivity but low specificity).

### Clinical outcomes

Seventy-two statistically significant clinical outcomes were reported, of which 90.3% (65/72) were positive, 6.9% (5/72) were negative, and 2.8% (2/72) were mixed (Table 3). Tables S1, S2, S3 and S4 in Additional file 1 illustrate the clinical outcomes specific to rounding, handover, CLABSI prevention and airway management checklists, respectively.

**Table 3:** Statistically significant clinical outcomes reported by articles (n)

Significant clinical outcomes reported	Positive/Improve	Negative/Worsen	Mixed
ICU length of stay	9	2	
Mechanical ventilation duration/use	8	1	1
CLABSI/CRBSI rate	8		
Urinary catheter duration/use	7		1
Central venous catheter duration/use	6	1	
Catheter-associated urinary tract infection/urinary tract infection rate	5		
Hospital length of stay	4	1	
Hospital mortality	3		
VAP/ventilator–associated events	3		
ICU mortality	2		
Reintubation rate	2		
Adverse intubation-associated events	2		
Accidental extubation	1		
28-day mortality	1		
ICU readmission rate	1		
Pneumonia	1		
Pulmonary embolism	1		
Infection rate	1		
Total	65	5	2

*CLABSI* Central line-associated bloodstream infection, *CRBSI* Catheter-related bloodstream infection, *VAP* Ventilator-associated pneumonia

#### Process of care outcomes

93.4% (114/122) of statistically significant process of care outcomes were positive, with the remaining 6.6% (8/122) identified as negative (Additional file 1: Table S5). 51% (26/51) of the unique process of care outcomes were by only one article. Tables S6, S7, S8 and S9 in Additional file 1 illustrate the process of care outcomes specific to rounding, handover, CLABSI prevention and airway management checklists, respectively.

### Checklist development, implementation, and validation

#### Type of study

Most articles on rounding, transfer/handover, CLABSI prevention and airway management checklists were implementation studies while delirium screening checklist articles were primarily validation studies (Additional file 1: Table S10). The remaining minority of studies primarily consisted of systematic reviews, meta-analyses, and surveys, which were conducted to varying degrees for each type of checklist.

With regard to the study design of the 123 primary articles, 55 (45%) articles were quasi-experimental studies, predominantly consisting of pre-post designs. Thirty-six (29%) were observational studies, most of which were delirium screening checklist articles. Five (4%) were randomised trials, including one randomised controlled trial (RCT) and one cluster RCT. The remaining 37 (30%) were systematic reviews and meta-analyses, surveys, and other unique study designs.

#### Checklist development

The method that authors used to develop the checklist studied in their article was investigated (Additional file 1: Table S11). All delirium screening checklist articles derived their checklist from existing literature. However, checklist development was highly variable among the other four checklist types. Development methodologies for these checklist types ranged were as follows: (1) not specified; (2) derived from existing literature; (3) already in existence at the institution; or (4) iteratively developed. Definitions for these terms are provided in the Additional file 1.

#### Concurrent interventions

We analysed the interventions that studies employed in conjunction with the checklists (Additional file 1: Table S12). Education or training was frequently used when introducing all checklist types. Feedback on performance or outcomes was limited and was mostly used in articles on CLABSI prevention checklists. Some patterns emerged when examining the most common “other” interventions used alongside certain checklist types. Rounding checklists were often paired with checklist champions (*n* = 5/47) and reminders (*n* = 6/47), transfer/handover checklists with handover protocols (*n* = 9/19), and CLABSI prevention checklists with central line insertion carts or trays (*n* = 3/8).

#### Use of post-intervention data

Post-intervention data were seldom used to improve or to modify the content of the checklists as this was evident in only eight articles.

### Guidelines

Ten guidelines were identified in the search. Three guidelines commented solely on delirium screening checklists, namely the ICDSC. Another two guidelines commented on the ICDSC along with procedural checklists, which included intubation as one of those procedures. Two guidelines provided recommendations for central line insertion checklists for the prevention of CLABSIs. Three guidelines provided guidance on intubation checklists, two of which were related to COVID-19 emergency intubation. No guidelines on rounding checklists or transfer/handover checklists were identified.

All five guidelines that mentioned delirium screening checklists recommended regular delirium screening using either the ICDSC or another common delirium screening tool, the Confusion Assessment Method for the Intensive Care Unit (CAM-ICU) [19–23]. In addition to recommending the use of the ICDSC, two of the guidelines required a checklist to be used for all invasive or high-risk procedures (including intubation) [22, 23].

Two guidelines recommended the implementation of any form of a central line insertion checklist in the ICU [24, 25]. Both provided an example of a central line insertion checklist from the Institute for Healthcare Improvement.

All three intubation checklist guidelines recommended the use of an intubation checklist. One provided an intubation checklist that was developed by its authors [26]. Two guidelines provided their recommendations in the context of COVID-19, each presenting a COVID-19 emergency intubation checklist [27, 28].

The 8-item ICDSC which was supported by the five guidelines has been repeatedly validated before and after the guidelines were published. All airway management checklist studies that provided information on the contents of their checklist on intubation were conducted before the earliest intubation checklist guidelines (since 2012). Accordingly, none of the intubation checklists published by the guidelines have had another study validate their efficacy.

### Checklists endorsed by major intensive care faculties and societies

Checklists identified and endorsed by major intensive care societies are listed below in Table 4 [29–35]. These checklists were analysed for whether they were developed in or from published literature or grey literature.

**Table 4:** Checklists endorsed by major intensive care faculties and societies

Faculties or societies	Checklist	Based on or found in literature
SCCM	Daily care rounding checklist [29]	Yes
ICS and FICM	Percutaneous tracheostomy checklist [30]	Yes
	Intubation checklist [30]	Yes
	Central line insertion checklist [30]	Yes
	Bronchoscopy checklist [30]	Yes
	Chest drain checklist [30]	Yes
	Nasogastric tube insertion checklist [30]	Yes
IHI	Central line insertion checklist [31]	No
	Ventilator bundle checklist [32]	No
	Daily goals checklist [33]	Yes
	Family contact checklist [34]	No
ANZICS	Central line insertion checklist [35]	No

*SCCM* Society of Critical Care Medicine, *ICS* Intensive Care Society, *FICM* Faculty of Intensive Care Medicine, *IHI* Institute for Health Improvement, *ANZICS* Australia and New Zealand Intensive Care Society

The SCCM daily care rounding checklist was not identified or mentioned in any literature; however, it was developed based on the recommendations identified in two SCCM publications [36, 37]. The six procedural checklists endorsed by ICS and FICM were developed and published in an editorial [38]. The IHI’s daily goals checklist was a modified version of the daily goals worksheet published in a 2003 article [39]. The other three IHI checklists were not identified in any literature; however, the central line insertion checklist was named as example checklist by the two CLABSI prevention guidelines included in this review [24, 25]. ANZICS’ central line insertion checklist had no information on its development.

## Discussion

This scoping review has identified an increasing use of checklists within the ICU. Five checklist categories were most frequently described and utilised during rounding, delirium screening, handover, CLABSI prevention and airway management. Although checklists are being used in ICU globally, 45% of research included in this study was published from the USA. Most checklists identified were physical checklists; however, use of electronic checklists has increased over the past 10 years. The length of most ICU checklists varied between six and 20 items, with checklists identified in guidelines being longer (averaging 41 items). The uptake of recommended checklists has been excellent, with the median adherence rate ranging from 73 to 91%. Significant clinical and process of care outcomes associated with checklist use that we identified were also overwhelmingly positive.

The ICDSC, a delirium screening checklist, was the most ubiquitous individual checklist in the ICU specific literature, as well as the most validated. Other checklist types appear to have vastly more implementation studies than validation data. Checklist development was highly variable. Most checklists were taken from a previous study (41%), although more than half these were delirium screening checklist studies. Following this, iterative development was the most common development process (20%) followed by those modified or adapted from previous institutional checklists before implementation (11%). In one of four checklists identified, the development process was not specified (28%).

Checklists were frequently implemented alongside education, training, or other interventions unique to the checklist studied. A considerable portion of articles failed to provide sufficient detail of their intervention, and as such, we opted not to apply the TIDieR tool to assess the reproducibility of the included papers’ interventions. Validation of some of the specific checklists identified is evident by current guidelines which support the use of the ICDSC, central line insertion checklists, procedural checklists, and intubation checklists. Unfortunately, few articles reported post-intervention data used to modify the checklist studied and improve its efficacy.

Physical checklists remain the most identified medium utilised, with an increasing use of the electronic checklist over the past decade demonstrated by this review. It is suspected that most of the checklist mediums that were not specified were likely physical checklists which would further emphasise the dominance of paper checklists in ICUs. Increasing trends in electronic media is possibly due to advancements capability or increased use of electronic medical records (EMR) [40]. As shown in this study, more dynamic checklists which draw on EMR data to allow patient specific modification is possible through the use of electronic media.

To our knowledge, this is the first published scoping review on the use and efficacy of checklists within the ICU but other reviews on related topics have previously been published [9, 10, 41–49]. We identified one other scoping review on quality improvement tools for the nursing care of long stay patients in the ICU [41]. Ten systematic reviews on specific checklists have been published since 2012 [9, 10, 42–49]. Six of the systematic reviews analysed delirium screening checklists specifically and one was published for each of the other four checklist types we identified. The results of these reviews mostly aligned with the outcomes of our scoping review. The systematic reviews on the ICDSC, a delirium screening checklist, were supportive of its use following the assessment of its validity [9, 42–46]. One systematic review on bedside ward round checklists found significant improvements in ICU length of stay (ICU LOS) and mechanical ventilation (MV) duration which were also the two most improved clinical outcomes identified by our scoping review [47]. The systematic reviews on handover checklists affirmed the improvements in information transfer and omissions found in our review [48]. One systematic review on CLABSI prevention interventions reported that checklists significantly reduced the risk of CLABSIs [10]. A systematic review on intubation checklists in critically ill patients did not find any significant outcomes in its ICU subgroup (*n* = 3 studies) while our review identified a few significant positive outcomes from the use of airway management checklists [49]. This scoping review significantly adds to the results from these reviews and also provides context around the development and implementation methods of the checklists, how they are currently used, and the clinical guidelines that recommend their use.

Interpreting the efficacy of checklists used in the ICU from the literature identified is a complex task. 72.5% (66/91) of articles on rounding, transfer/handover, CLABSI prevention, and airway management checklists found at least one statistically significant outcome. 27.5% (25/91) of these articles did not report a significant outcome. Of the 25 articles, seven measured for significance but did not report any significant change in outcomes. The remaining 18 articles did not report a significant clinical or process of care outcome due to the study design and/or statistical analysis employed by the researchers. Some studies (e.g. surveys or qualitative studies) did not measure any clinical outcomes. Other studies measured clinical or process of care outcomes but did not assess their significance. Accordingly, the lack of significant outcomes produced by these studies should not be interpreted as a failure of checklists to improve outcomes. There was also a degree of heterogeneity in the outcomes measured by articles both within checklist types and between checklist types. For example, one rounding checklist study would measure ICU LOS, MV duration, and CLABSI rate while another rounding checklist study would measure central venous catheter duration. This can prevent the identification of positive clinical outcomes associated with any intervention. Underpowering was also cited by some researchers to have contributed to the lack of significant outcomes in their respective studies.

When checklists adherence was low, it was usually due to clinicians’ choice not to use the checklist. Clinicians may feel a checklist is not useful, either to a specific patient or in general. Other explanations for low adherence include a short duration of adherence measurement, patients being discharged before checklist use, a lack of training or performance feedback being adopted, or a lack of “checklist champions”. Failure to achieve uptake by clinicians is a multifactorial problem, but one which may be further contributed to by the major intensive care societies. Many checklists identified in our scoping review of the published literature were not endorsed by multiple societies. We identified 12 checklists endorsed by the major societies, with limited published literature on either their development, implementation, or efficacy. Three of these were central line insertion checklists, two rounding checklists and seven others focused on unique tasks such as nasogastric tube insertion and intubation. This degree of variation in checklists endorsement by major intensive care societies creates confusion amongst clinicians. COVID-19 demonstrated that universal endorsement of airway management checklists and guidelines by professional societies and specialist colleges led to widespread adoption by practitioners [50]. There is a strong argument that the universal adoption of the best checklist by these societies would significantly improve their use within the clinical environment.

### Limitations

Scoping reviews are an excellent methodology for mapping out previous research findings and identifying the focus of future research relating to a broad topic. This review has focused on how checklists are utilised within the complex ICU environment [51]. However, scoping reviews are limited in their ability to comment on the outcomes of included papers because they do not assess the quality of evidence [51]. The breadth of literature covered in this review was pleasing, as was the depth of analysis, especially with mapping the statistically significant clinical outcomes related to individual checklist use. Another limitation to acknowledge in all review articles relates to database search strategy. Especially with a common term such as “checklists”. We limited our search for the term ‘checklist’ in the title of articles and Medical Subject Headings (MeSH) terms. To be confident that we have identified most of the literature on the use of checklists in the ICU from the last 10 years, we then utilised an extensive grey literature search and search of all references in included full-text articles. We limited our date range from 2012, excluded non-English articles, and were unable to access all full-text articles which may also have meant some important research was excluded. Some relevant articles may also have been missed during study selection due to only 10% of samples being double screened with two reviewers. A protocol for this scoping review was not registered which poses an additional limitation for this scoping review.

### Recommendations

Our scoping review demonstrates widespread use of checklists within the ICU. Future research should focus on our following recommendations:

1. More research is needed into the clinical outcomes associated with the use of checklists for both CLABSI prevention and airway management within the ICU.
2. Guidelines for rounding and handover within the ICU should consider adopting one specific checklist.
3. The main ICU societies should aim to achieve consensus regarding the specific checklists they endorse, hopefully in an evidence-based manner.
4. More RCTs are required to provide more robust evidence for all ICU checklist types.
5. Less heterogeneity in outcomes measures should be adopted in future research into specific checklists. It would be beneficial to establish which clinical and process of care outcomes are most important for each specific checklist.
6. A process of checklist modification using the post-intervention data should be adopted for each endorsed checklist.
7. Dynamic, electronic checklists may be the best medium to utilise to allow the above recommendations to occur.

## Conclusion

Within the ICU, checklists are widely used and now included in many clinical guidelines. They are also generally well implemented and validated, most especially in delirium screening. Most research into clinical outcomes associated with checklist use in the ICU is overwhelmingly positive. Future research on checklists within the ICU should now focus on how they appear in clinical guidelines, which ones should be universally endorsed and allow for a process of user-checklist feedback for ongoing checklist modification and improvement.

### Supplementary Information

**Additional file 1.** Online supporting information.

## Data Availability

The datasets used and/or analysed during the current study are available from the corresponding author on reasonable request.

## Abbreviations

ICU: Intensive care unit
CLABSI: Central line-associated bloodstream infection
RQ: Research question
ICDSC: Intensive care delirium screening checklist
CRBSI: Catheter-related bloodstream infection
EHR: Electronic health record
RCT: Randomised controlled trial
CAM-ICU: Confusion Assessment Method for the Intensive Care Unit
SCCM: Society of Critical Care Medicine
ICS: Intensive Care Society
FICM: Faculty of Intensive Care Medicine
IHI: Institute for Health Improvement
ANZICS: Australia and New Zealand Intensive Care Society
LOS: Length of stay
MV: Mechanical ventilation
