# Supplementary material for: The use of checklists in the intensive care unit: a scoping review
Source: Crit Care. 2023 Nov 30;27:468. doi: 10.1186/s13054-023-04758-2 (PMC10691022; doi:10.1186/s13054-023-04758-2)

Online Supporting Information

Methods. Syntaxes Used in Database Searches

Appendix S1. Major Intensive Care Societies

Appendix S2. List of Included Articles

Table S1. Statistically Significant Clinical Outcomes Reported by Rounding Checklist Articles (n)

Table S2. Statistically Significant Clinical Outcomes Reported by Transfer & Handover Checklist Articles (n)

Table S3. Statistically Significant Clinical Outcomes Reported by CLABSI Prevention Checklist Articles (n)

Table S4. Statistically Significant Clinical Outcomes Reported by Airway Management Checklist Articles (n)

Table S5. Statistically Significant Process of Care Outcomes Reported by Articles (n)

Table S6. Statistically Significant Process of Care Outcomes Reported by Rounding Checklist Articles (n)

Table S7. Statistically Significant Process of Care Outcomes Reported by Transfer & Handover Checklist Articles (n)

Table S8. Statistically Significant Process of Care Outcomes Reported by CLABSI Prevention Checklist Articles (n)

Table S9. Statistically Significant Process of Care Outcomes Reported by Airway Management Checklist Articles (n)

Table S10. Articles by Type of Study

Table S11. Checklist Development Method

Table S12. Concurrent Interventions

Figure S1. Articles by Region (n)

Figure S2. Articles by Year (n)

Figure S3. Length of Checklists

Figure S4. Articles Using Electronic Checklists

Figure S5. Articles Using Physical Checklists

Methods. Syntax Used in Database Searches

Ovid (Medline and Embase)

((intensive care* OR critical care* OR ICU OR ICUs OR ((intensive or critical) adj2 care*)).mp. OR exp intensive care unit/ OR exp intensive care/) AND (((checklist*).mp. AND *checklist/) OR (ticklist* OR tick list* OR checksheet* OR check sheet* OR goalsheet* OR goal sheet*).mp. OR (intubation checklist OR central line insertion checklist OR intensive care delirium screening checklist OR ICDSC OR percutaneous tracheostomy checklist OR bronchoscopy checklist OR chest drain checklist OR nasogastric tube insertion checklist OR FASTHUG* OR round* checklist OR discharge checklist).mp. OR (checklist* or check list*).ti.)
Limits: english language AND yr="2012-current"

Scopus

(TITLE ("checklist*" OR "check list*" OR "ticklist*" OR "tick list*" OR "checksheet*" OR "check sheet*" OR "goalsheet*" OR "goal sheet*") AND TITLE-ABS-KEY ("intensive care" OR "critical care" OR "ICU" OR "ICUs" OR ("intensive" PRE/2 "care"))) AND PUBYEAR > 2011 AND (LIMIT-TO (LANGUAGE , "English"))

Google Scholar

allintitle: ("checklist" OR "check list" OR "ticklist" OR "tick list" OR "goalsheet" OR "goal sheet") AND ("ICU" OR "intensive care" OR "critical care" OR "ICUs")

Appendix S1. Major Intensive Care Societies

- Society of Critical Care Medicine (SCCM)
- Intensive Care Society (ICS)
- Institute for Health Improvement (IHI)
- Faculty of Intensive Care Medicine (FICM)
- Australia and New Zealand Intensive Care Society (ANZICS)
- European Society of Critical Care Medicine (ESICM)
- College of Intensive Care Medicine (CICM)
- Neurocritical Care Society (NCS)

Appendix S2. List of Included Articles

Title	Year	Author
Use of a checklist and clinical decision support tool reduces laboratory use and improves cost.	2016	Algaze et al.
Systematic review and meta-analysis of interventions for operating room to intensive care unit handoffs.	2021	Abraham et al.
Effect of compliance with a nurse-led intensive care unit checklist on clinical outcomes in mechanically and nonmechanically ventilated patients.	2016	Al Ashry et al.
Effect of daily use of electronic checklist on physical rehabilitation consultations in critically ill patients.	2017	Ali et al.
Informing the standardising of care for prolonged stay patients in the intensive care unit: a scoping review of quality improvement tools.	2022	Allum et al.
Arabic intensive care delirium screening checklist's validity and reliability: a multicenter study.	2019	Al-Qadheeb et al.
Improving patient safety: usefulness of safety checklists in a neonatal unit.	2017	Arriaga Redondo et al.
Use of a nursing checklist to facilitate implementation of therapeutic hypothermia after cardiac arrest.	2015	Avery et al.
Impact of a multidisciplinary checklist on the duration of invasive mechanical ventilation and length of ICU stay.	2020	Barcellos et al.
Diagnostic accuracy of delirium assessment methods in critical care patients.	2018	Barman et al.
Evidence and consensus based guideline for the management of delirium, analgesia, and sedation in intensive care medicine. Revision 2015 (DAS-Guideline 2015)–short version.	2015	Baron et al.
Clinical practice guidelines for the management of pain, agitation, and delirium in adult patients in the intensive care unit.	2013	Barr et al.
Prevention of central line–associated bloodstream infections through quality improvement interventions: a systematic review and meta-analysis.	2014	Blot et al.
Decreasing failed extubations with the implementation of an extubation checklist.	2019	Bobbs et al.
Delirium in the intensive care setting: a reevaluation of the validity of the CAM–ICU and ICDSC versus the DSM–IV–TR in determining a diagnosis of delirium as part of the daily clinical routine.	2017	Boettger et al.
Screening for delirium with the Intensive Care Delirium Screening Checklist (ICDSC): a re-evaluation of the threshold for delirium.	2018	Boettger et al.
Screening for delirium with the Intensive Care Delirium Screening Checklist (ICDSC): symptom profile and utility of individual items in the identification of delirium dependent on the level of sedation.	2019	Boettger et al.
Documentation in a PICU setting: is a checklist tool effective?	2012	Boucher et al.
Use of a standardized care communication checklist during multidisciplinary rounds in pediatric cardiac intensive care: a best practice implementation project.	2018	Boydston.
Consensus statement: Safe Airway Society principles of airway management and tracheal intubation specific to the COVID-19 adult patient group.	2020	Brewster et al.
Strategies to prevent central line-associated bloodstream infections in acute-care hospitals: 2022 update.	2022	Buetti et al.
Intensive care unit rounding checklist implementation. Effect of accountability measures on physician compliance.	2015	Carlos et al.
Improving compliance with a rounding checklist through low-and high-technology interventions: a quality improvement initiative.	2021	Carr et al.
Delirium rating scales in critically ill patients: a systematic literature review.	2013	Carvalho et al.
Effect of a quality improvement intervention with daily round checklists, goal setting, and clinician prompting on mortality of critically ill patients: a randomized clinical trial.	2016	Cavalcanti.
Use of a daily goals checklist for morning ICU rounds: a mixed-methods study.	2014	Centofanti et al.

Quality improvement in the trauma intensive care unit using a rounding checklist: the implementation results.	2017	Chang et al.
The 2014 updated version of the Confusion Assessment Method for the Intensive Care Unit compared to the 5th version of the Diagnostic and Statistical Manual of Mental Disorders and other current methods used by intensivists.	2018	Chanques et al.
Diagnostic accuracy of the CAM-ICU and ICDSC in detecting intensive care unit delirium: a bivariate meta-analysis.	2021	Chen et al.
Safety first! Using a checklist for intrafacility transport of adult intensive care patients.	2015	Comeau et al.
Validating a process-of-care checklist for intensive care units.	2013	Conroy et al.
Testing the implementation of an electronic process-of-care checklist for use during morning medical rounds in a tertiary intensive care unit: a prospective before-after study.	2015	Conroy et al.
A national survey of the impact of NAP4 on airway management practice in United Kingdom hospitals: closing the safety gap in anaesthesia, intensive care and the emergency department.	2016	Cook et al.
Consensus guidelines for managing the airway in patients with COVID-19: guidelines from the Difficult Airway Society, the Association of Anaesthetists the Intensive Care Society, the Faculty of Intensive Care Medicine and the Royal College of Anaesthetists.	2020	Cook et al.
Structured handoff checklists improve clinical measures in patients discharged from the neurointensive care unit.	2015	Coon et al.
Intelligent checklists improve checklist compliance in the intensive care unit: a prospective before-and-after mixed-method study.	2021	De Bie et al.
Psychometric properties of the intensive care delirium screening checklist when used by bedside nurses in clinical practice: a prospective descriptive study.	2020	Detroyer et al.
Clinical practice guidelines for the prevention and management of pain, agitation/sedation, delirium, immobility, and sleep disruption in adult patients in the ICU.	2018	Devlin et al.
A standard handoff improves cardiac surgical patient transfer: operating room to intensive care unit.	2015	Dixon et al.
Cultural and linguistic validation of the Italian version of the intensive care delirium screening checklist.	2012	Domenico et al.
A pediatric intensive care checklist for Interprofessional rounds: the R-PICniC study.	2022	Dos Santos Alves et al.
Implementation of an electronic checklist in the ICU: association with improved outcomes.	2018	Duclos et al.
Implementation of systematic safety checklists in a neurocritical care unit: a quality improvement study.	2022	Escamilla-Ocanas et al.
Implementation of a checklist to increase adherence to evidence-based practices in a single pediatric intensive care unit.	2017	Eulmesekian et al.
An exploration of the reliability and usability of two delirium screening tools in an Australian intensive care unit: a pilot study.	2021	Ewers et al.
Beyond the bundle-journey of a tertiary care medical intensive care unit to zero central line-associated bloodstream infections.	2013	Exline et al.
Guidelines for the Provision of Intensive Care Services - version 2.	2019	Faculty of Intensive Care Medicine.
Guidelines for the Provision of Intensive Care Services - version 2.1.	2022	Faculty of Intensive Care Medicine.
CAM-ICU and ICDSC agreement in medical and surgical ICU patients is influenced by disease severity.	2012	Fagundes et al.
OR to ICU handoff: theory of change model for sustainable change in behavior.	2019	Faiz et al.
The effectiveness of a bundle in the prevention of ventilator-associated pneumonia.	2016	Ferreira et al.

Development and pilot of a checklist for management of acute liver failure in the intensive care unit.	2016	Fix et al.
Paediatric intensive care and neonatal intensive care airway management in the United Kingdom: the PIC-NIC survey.	2018	Foy et al.
An intervention to improve the catheter associated urinary tract infection rate in a medical intensive care unit: direct observation of catheter insertion procedure.	2017	Galiczewski et al.
A cohort study on nurse-led checklist intervention to reduce catheter-related bloodstream infection in an intensive care unit.	2017	Gameiro et al.
WE CARE 4 KIDS: use of a rounding tool in the pediatric intensive care unit.	2017	Ganesan et al.
Implementing rounding checklists in a pediatric oncologic intensive care unit.	2022	Gardner et al.
Delirium assessment tools for use in critically ill adults: a psychometric analysis and systematic review.	2018	Gelinas et al.
Impact of a delirium screening tool and multifaceted education on nurses' knowledge of delirium and ability to evaluate it correctly.	2012	Gesin et al.
eSIMPLER: a dynamic, electronic health record–integrated checklist for clinical decision support during PICU daily rounds.	2021	Geva et al.
Improving cardiac operating room to intensive care unit handover using a standardised handover process.	2017	Gleicher et al.
Improving the quality of radiographs in neonatal intensive care unit utilizing educational interventions.	2015	Gupta et al.
The confusion assessment method for the intensive care unit (CAM-ICU) and intensive care delirium screening checklist (ICDSC) for the diagnosis of delirium: a systematic review and meta-analysis of clinical studies.	2012	Gusmao-Flores et al.
Effect of sedation level on the prevalence of delirium when assessed with CAM-ICU and ICDSC.	2013	Haenggi et al.
Perceptions of rounding checklists in the intensive care unit: a qualitative study.	2018	Hallam et al.
Interventions to improve patient safety during intubation in the neonatal intensive care unit.	2016	Hatch et al.
The impact of a multidisciplinary safety checklist on adverse procedural events during bedside bronchoscopy-guided percutaneous tracheostomy.	2015	Hazelton et al.
Before-after study of a checklist to improve acute care to ICU handoffs.	2023	Hicks et al.
Guidelines for the management of tracheal intubation in critically ill adults.	2018	Higgs et al.
Diagnostic performance of delirium assessment tools in critically ill patients: a systematic review and meta-analysis.	2020	Ho et al.
The Valproate Checklist in a female psychiatric intensive care unit.	2018	Howe et al.
Design and α-testing of an electronic rounding tool (CERTAINp) to improve process of care in pediatric intensive care unit.	2017	Hulyalkar et al.
Removing nonessential central venous catheters: evaluation of a quality improvement intervention.	2012	Ilan et al.
Performance checklist and its influence on knowledge and satisfaction of intensive care nurses: a quasi-experimental study.	2023	Imanipour et al.
Current status of delirium assessment tools in the intensive care unit: a prospective multicenter observational survey.	2022	Ishii et al.
A multicenter randomized trial of a checklist for endotracheal intubation of critically ill adults.	2018	Janz et al.
Implementation of the comprehensive unit-based safety program to improve infection prevention and control practices in four neonatal intensive care units in Pune, India.	2022	Johnson et al.
The "TRAUMA LIFE" initiative: the impact of a multidisciplinary checklist process on outcomes and communication in a trauma intensive care unit.	2018	Joseph et al.
Improving the timeliness and efficiency of discharge from the NICU.	2022	Kaemingk et al.
The effect of a quality improvement intervention on perceived sleep quality and cognition in a medical ICU.	2013	Kamdar et al.

Implementation of a structured information transfer checklist improves postoperative data transfer after congenital cardiac surgery.	2013	Karakaya et al.
A multidisciplinary handoff process to standardize the transfer of care between the intensive care unit and the operating room.	2018	Karamchandani et al.
Effectiveness of a daily rounding checklist on processes of care and outcomes in diverse pediatric intensive care units across the world.	2021	Kashyap et al.
A methodological study to develop a 'nursing checklist' for receiving patients in ICUs.	2015	Kaushal et al.
Adding value to daily chest X-rays in the ICU through education, restricted daily orders and indication-based prompting.	2017	Keveson et al.
Reliability and validity of the checklist for early recognition and treatment of acute illness and injury as a charting tool in the medical intensive care unit.	2017	Kogan et al.
Reliability and validity of the Intensive Care Delirium Screening Checklist in Turkish.	2016	Kose et al.
The CAM-ICU-7 and ICDSC as measures of delirium severity in critically ill adult patients.	2020	Krewulak et al.
Delirium assessment in neuro-critically ill patients: a validation study.	2019	Larsen et al.
Integrating a safety smart list into the electronic health record decreases intensive care unit length of stay and cost.	2020	Lemkin et al.
Development, validation and application of a ventilator-associated pneumonia prevention checklist in a single cardiac surgery centre.	2018	Li et al.
Predicting hospital mortality and length of stay: a prospective cohort study comparing the Intensive Care Delirium Screening Checklist versus Confusion Assessment Method for the Intensive Care Unit.	2023	Li et al.
Improving handoffs between operating room and pediatric intensive care teams: before and after study.	2018	Malenka et al.
Impact of enhanced ventilator care bundle checklist on nursing documentation in an intensive care unit.	2013	Malouf-Todaro et al.
The impact of daily use of an enteral feeding checklist on clinical outcomes in shock patients: a retrospective cohort study.	2019	Mao et al.
Effects of multidisciplinary rounds and checklist in an intensive care unit: a mixed methods study.	2022	Maran et al.
Multiprofessional round with checklist: association with the improvement in patient safety in intensive care.	2022	Maran et al.
Strategies to prevent central line-associated bloodstream infections in acute care hospitals: 2014 update.	2014	Marschall et al.
Handover from operating theatre to the intensive care unit: a quality improvement study.	2019	Marshall et al.
A comfort measures only checklist for critical care providers: impact on satisfaction and symptom management.	2021	Matone et al.
Using a post-intubation checklist and time out to expedite mechanical ventilation monitoring: observational study of a quality improvement intervention.	2016	McConnell et al.
A PICU patient safety checklist: rate of utilization and impact on patient care.	2016	McKelvie et al.
Central line insertion bundle: experiences and challenges in an adult ICU.	2012	McPeake et al.
Long-term prevention of catheter-associated urinary tract infections among critically ill patients through the implementation of an educational program and a daily checklist for maintenance of indwelling urinary catheters: a quasi-experimental study.	2019	Menegueti et al.
Multipronged strategy to reduce routine-priority blood testing in intensive care unit patients.	2016	Merkeley et al.
Organizational factors associated with adherence to low tidal volume ventilation: a secondary analysis of the CHECKLIST-ICU database.	2020	Midega et al.
Delirium diagnosis without a gold standard: evaluating diagnostic accuracy of combined delirium assessment tools.	2022	Moss et al.

Implementation of a standardized handoff protocol for post-operative admissions to the surgical intensive care unit.	2018	Mukhopadhyay et al.
A standardized checklist improves the transfer of stroke patients from the neurocritical care unit to hospital ward.	2020	Murray et al.
Influence of sedation level and ventilation status on the diagnostic validity of delirium screening tools in the ICU—an international, prospective, bi-center observational study (IDeAS).	2020	Nacul et al.
Impact of multiprofessional rounds on critical care outcomes in the surgical trauma intensive care unit.	2019	Nahouraii et al.
Successful introduction of a daily checklist to enhance compliance with accepted standards of care in the medical intensive care unit.	2016	Nama et al.
Intensive care unit rounding checklists to reduce catheter-associated urinary tract infections.	2020	Nassikas et al.
COVID-19 screening and testing among patients with neurologic dysfunction: the neuro-COVID-19 time-out process and checklist.	2020	Natteru et al.
Delirium screening in critically ill patients: a systematic review and meta-analysis.	2012	Neto et al.
Checklists change communication about key elements of patient care.	2012	Newkirk et al.
A pragmatic checklist to identify pediatric ICU patients at risk for cardiac arrest or code bell activation.	2016	Niles et al.
Sensitivity and specificity of the Confusion Assessment Method for the Intensive Care Unit (CAM-ICU) and the Intensive Care Delirium Screening Checklist (ICDSC) for detecting post-cardiac surgery delirium: a single-center study in Japan.	2016	Nishimura et al.
Sustained improvement in tracheal intubation safety across a 15-center quality-improvement collaborative: an interventional study from the National Emergency Airway Registry for Children Investigators.	2021	Nishisaki et al.
The use of nurse checklists in a bedside computer-based information system to focus on avoiding secondary insults in neurointensive care.	2012	Nyholm et al.
Use of electronic medical record-enhanced checklist and electronic dashboard to decrease CLABSIs.	2014	Pageler et al.
Pilot implementation of a perioperative protocol to guide operating room-to-intensive care unit patient handoffs.	2012	Petrovic et al.
Analysis of daily goal sheets on physician-nurse collaboration attitude.	2020	Qian et al.
Reduction of laboratory utilization in the intensive care unit.	2017	Raad et al.
Evaluation of a paper-based checklist versus an electronic handover tool based on the situation background assessment recommendation (SBAR) concept in patients after surgery for congenital heart disease.	2021	Rehm et al.
Use of a bundle checklist combined with physician confirmation reduces risk of nosocomial complications and death in trauma patients compared to documented checklist use alone.	2015	Reiff et al.
User requirements analysis of the Intelligent Intensive Care Delirium Screening Checklist.	2018	Ren et al.
Deconstructing poststroke delirium in a prospective cohort of patients with intracerebral hemorrhage.	2020	Reznik et al.
Reducing the rate of catheter-associated bloodstream infections in a surgical intensive care unit using the Institute for Healthcare Improvement Central Line Bundle.	2014	Sacks et al.
Safety checklist implementation did not reduce central venous catheter duration in pediatric cardiac ICU patients.	2020	Sahulee et al.
The effect of a checklist on the quality of patient handover from the operating room to the intensive care unit: a randomized controlled trial.	2016	Salzwedel et al.
'Safety by DEFAULT': introduction and impact of a paediatric ward round checklist.	2013	Sharma et al.
Introduction of a new ward round approach in a cardiothoracic critical care unit.	2015	Shaughnessy et al.
Impact of a daily PICU rounding checklist on urinary catheter utilization and infection.	2018	Siegel et al.

Use of bundled interventions, including a checklist to promote compliance with aseptic technique, to reduce catheter-related bloodstream infections in the intensive care unit.	2014	Simpson et al.
Intensive care unit readmission prevention checklist: is it worth the effort?	2014	Smischney et al.
Organizational characteristics, outcomes, and resource use in 78 Brazilian intensive care units: the ORCHESTRA study.	2015	Soares et al.
Standardization of postoperative transitions of care to the pediatric intensive care unit enhances efficiency and handover comprehensiveness.	2016	Sochet et al.
An individualized recovery task checklist which served as an educational instrument in a critically ill and intubated COVID-19 patient.	2022	Stantz et al.
Improving infant vaccination status in a level IV neonatal intensive care unit.	2019	Stetson et al.
Antibiotic surveillance on a paediatric intensive care unit: easy attainable strategy at low costs and resources.	2012	Stocker et al.
Introduction of a rounding sticker improves care and reduces infection rates in the pediatric intensive care unit (PICU).	2012	Stroud et al.
A quality improvement initiative to reduce central line infection in neonates using checklists.	2017	Taylor et al.
Measurable outcomes of quality improvement using a daily quality rounds checklist: two-year prospective analysis of sustainability in a surgical intensive care unit.	2013	Teixeira et al.
Comparison of CAM-ICU and ICDSC for the detection of delirium in critically ill patients focusing on relevant clinical outcomes.	2012	Tomasi et al.
The effect of a quality improvement intervention on sleep and delirium in critically ill patients in a surgical ICU.	2021	Tonna et al.
Using checklists and repetitive simulation to improve patient safety: a pilot project with the Impella Left Ventricular Assist Device.	2017	Turkelson et al.
Improving communication between surgery and critical care teams: beyond the handover.	2018	Turner et al.
Association of checklist use in endotracheal intubation with clinically important outcomes: a systematic review and meta-analysis.	2020	Turner et al.
Let the EHR talk loudly: an EHR-connected verbal surgical safety checklist for medical procedures in the intensive care unit.	2022	Uppot et al.
Do digital handover checklists influence the clinical outcome parameters of intensive care unit patients? A randomized controlled pilot study.	2021	Verholen et al.
Delirium screening in neurocritical care and stroke unit patients: a pilot study on the influence of neurological deficits on CAM-ICU and ICDSC outcome.	2020	von Hofen-Hohloch et al.
Checklist for early recognition and treatment of acute illness and injury: an exploratory multicenter international quality-improvement study in the ICUs with variable resources.	2021	Vukoja et al.
The design and application of an intensive care unit point-of-care nursing handover checklist based on the situation, background, assessment, and recommendation technique.	2022	Wang et al.
Empiric antibiotic, mechanical ventilation, and central venous catheter duration as potential factors mediating the effect of a checklist prompting intervention on mortality: an exploratory analysis.	2012	Weiss et al.
A clinical trial comparing physician prompting with an unprompted automated electronic checklist to reduce empirical antibiotic utilization.	2013	Weiss et al.
Prompting physicians to address a daily checklist for antibiotics: do we need a co-pilot in the ICU?	2013	Weiss et al.
Influence of patient-specific covariates on test validity of two delirium screening instruments in neurocritical care patients (DEMON-ICU).	2022	Weiss et al.
Systematic evaluation of the effect of bedside ward round checklist on clinical outcomes of critical patients.	2021	Wen et al.
Efficacy of introducing a checklist to reduce central venous line associated bloodstream infections in the ICU caring for adult patients.	2018	Wichmann et al.
A checklist for intrahospital transport of critically ill patients improves compliance with transportation safety guidelines.	2020	Williams et al.

Long-term persistence of quality improvements for an intensive care unit communication initiative using the VALUE strategy.	2014	Wysham et al.
Improving the postoperative handover process in the intensive care unit of a tertiary teaching hospital.	2016	Yang et al.
Laboratory tests and x-ray imaging in a surgical intensive care unit: checking the checklist.	2018	Yorkgitis et al.
Evaluating pain, sedation, and delirium in the neurologically critically ill-feasibility and reliability of standardized tools: a multi-institutional study.	2013	Yu et al.
Compliance with prevention practices and their association with central line-associated bloodstream infections in neonatal intensive care units.	2014	Zachariah et al.
Application effect of humanistic care time checklist on nursing quality of ICU patients.	2023	Zou et al.

Table S1. Statistically Significant Clinical Outcomes Reported by Rounding Checklist Articles (n)

Significant Clinical Outcomes Reported	Positive/Improve	Negative/Worsen	Mixed
ICU length of stay	9	1	-
Mechanical ventilation duration/use	7	1	1
CLABSI/CRBSI rate	1	-	-
Urinary catheter duration/use	6	-	1
Central venous catheter duration/use	6	1	-
Catheter-associated urinary tract infection/urinary tract infection rate	5	-	-
Hospital length of stay	3	1	-
Hospital mortality	3	-	-
VAP/ventilator-associated events	3	-	-
ICU mortality	2	-	-
Reintubation rate	1	-	-
Adverse intubation-associated events	-	-	-
Accidental extubation	1	-	-
28-day mortality	1	-	-
ICU readmission rate	-	-	-
Pneumonia	1	-	-
Pulmonary embolism	1	-	-
Infection rate	1	-	-
Total	51	4	2

Table S2. Statistically Significant Clinical Outcomes Reported by Transfer & Handover Checklist Articles (n)

Significant Clinical Outcomes Reported	Positive/Improve	Negative/Worsen	Mixed
ICU length of stay	-	-	-
Mechanical ventilation duration/use	1	-	-
CLABSI/CRBSI rate	-	-	-
Urinary catheter duration/use	1	-	-
Central venous catheter duration/use	-	-	-
Catheter-associated urinary tract infection/urinary tract infection rate	-	-	-
Hospital length of stay	1	-	-
Hospital mortality	-	-	-
VAP/ventilator-associated events	-	-	-
ICU mortality	-	-	-
Reintubation rate	-	-	-
Adverse intubation-associated events	-	-	-
Accidental extubation	-	-	-
28-day mortality	-	-	-
ICU readmission rate	1	-	-
Pneumonia	-	-	-
Pulmonary embolism	-	-	-
Infection rate	-	-	-
Total	4	0	0

Table S3. Statistically Significant Clinical Outcomes Reported by CLABSI Prevention Checklist Articles (n)

**Significant Clinical Outcomes Reported**	**Positive/Improve**	**Negative/Worsen**	**Mixed**
ICU length of stay	-	-	-
Mechanical ventilation duration/use	-	-	-
CLABSI/CRBSI rate	7	-	-
Urinary catheter duration/use	-	-	-
Central venous catheter duration/use	-	-	-
Catheter-associated urinary tract infection/urinary tract infection rate	-	-	-
Hospital length of stay	-	-	-
Hospital mortality	-	-	-
VAP/ventilator-associated events	-	-	-
ICU mortality	-	-	-
Reintubation rate	-	-	-
Adverse intubation-associated events	-	-	-
Accidental extubation	-	-	-
28-day mortality	-	-	-
ICU readmission rate	-	-	-
Pneumonia	-	-	-
Pulmonary embolism	-	-	-
Infection rate	-	-	-
Total	7	0	0

Table S4. Statistically Significant Clinical Outcomes Reported by Airway Management Checklist Articles (n)

**Significant Clinical Outcomes Reported**	**Positive/Improve**	**Negative/Worsen**	**Mixed**
ICU length of stay	-	-	-
Mechanical ventilation duration/use	1	-	-
CLABSI/CRBSI rate	-	-	-
Urinary catheter duration/use	1	-	-
Central venous catheter duration/use	-	-	-
Catheter-associated urinary tract infection/urinary tract infection rate	-	-	-
Hospital length of stay	1	-	-
Hospital mortality	-	-	-
VAP/ventilator-associated events	-	-	-
ICU mortality	-	-	-
Reintubation rate	-	-	-
Adverse intubation-associated events	-	-	-
Accidental extubation	-	-	-
28-day mortality	-	-	-
ICU readmission rate	1	-	-
Pneumonia	-	-	-
Pulmonary embolism	-	-	-
Infection rate	-	-	-
Total	4	0	0

Table S5. Statistically Significant Process of Care Outcomes Reported by Articles (n)

Significant Process of Care Outcomes	Positive/Improve	Negative/Worsen	Mixed
Information transfer/sharing	10	-	-
Attendance	7	-	-
Sedation/sedation management	5	2	-
DVT/VTE prophylaxis	5	1	-
Low tidal volume ventilation/lung protective ventilation	5	-	-
Stress ulcer prophylaxis/ peptic ulcer prophylaxis	5	-	-
Omissions	5	-	-
Medication review/ reconciliation/ adjustments	5	-	-
Head of bed elevation	4	-	-
Central venous catheter removal/need assessment	4	-	-
Lab utilisation	4	-	-
Round/handover completion time	3	1	-
Spontaneous breathing trial	3	-	-
Pain/pain management	3	-	-
Glucose management	3	-	-
Antimicrobial/ antibiotic use	2	1	-
Antimicrobial/ antibiotic need assessment	2	-	-
Nutrition assessment	2	-	-
Weight	2	-	-
Urinary catheter removal/need assessment	2	-	-
Lab necessity/ diagnostics	2	-	-
Family updates/ communication	2	-	-
Occupational therapy/ physical therapy	2	-	-
Bowel protocol/ movement	2	-	-
Mechanical ventilation weaning, extubation planning/readiness	1	1	-

Table S5. Statistically Significant Process of Care Outcomes Reported by Articles (n) (continued)

Significant Process of Care Outcomes	Positive/Improve	Negative/Worsen	Mixed
Endotracheal tube need assessment	1	-	-
Medication by mouth	1	-	-
Association between checklist responses and care delivered	1	-	-
Care coordination	1	-	-
Electrolytes	1	-	-
Goals of care	1	-	-
Fluid balance	1	-	-
Skin/wound care	1	-	-
Infection prevention	1	-	-
Need for devices	1	-	-
Central venous catheter dressing, cap, port needle change frequency	1	-	-
Daily oral care	1	-	-
Turning patient (pressure ulcer prevention)	1	-	-
Technical errors	1	-	-
Time to analgesia dosing	1	-	-
Faster ABGs after intubation	1	-	-
Concerns on rounds	1	-	-
Oxygenation index	1	-	-
Tracheostomy	1	-	-
Restraint order compliance	1	-	-
Hospital costs	1	-	-
Handover occurs at ICU bedside	1	-	-
Adequate handover preparation	1	-	-
Address patient's treatment preferences	1	-	-
Central venous catheter insertion bundle documentation	-	1	-
Head circumference measurement	-	1	-
**Total**	**114**	**8**	**0**

Table S6. Statistically Significant Process of Care Outcomes Reported by Rounding Checklist Articles (n)

**Significant Process of Care Outcomes**	**Positive/Improve**	**Negative/Worsen**	**Mixed**
Information transfer/sharing	-	-	-
Attendance	2	-	-
Sedation/sedation management	5	2	-
DVT/VTE prophylaxis	5	1	-
Low tidal volume ventilation/lung protective ventilation	5	-	-
Stress ulcer prophylaxis/ peptic ulcer prophylaxis	5	-	-
Omissions	1	-	-
Medication review/ reconciliation/ adjustments	4	-	-
Head of bed elevation	4	-	-
Central venous catheter removal/need assessment	3	-	-
Lab utilisation	4	-	-
Round/handover completion time	1	1	-
Spontaneous breathing trial	3	-	-
Pain/pain management	3	-	-
Glucose management	3	-	-
Antimicrobial/ antibiotic use	2	1	-
Antimicrobial/ antibiotic need assessment	2	-	-
Nutrition assessment	2	-	-
Weight	2	-	-
Urinary catheter removal/need assessment	2	-	-
Lab necessity/ diagnostics	2	-	-
Family updates/ communication	2	-	-
Occupational therapy/ physical therapy	2	-	-
Bowel protocol/ movement	2	-	-
Mechanical ventilation weaning, extubation planning/readiness	1	1	-

Table S6. Statistically Significant Process of Care Outcomes Reported by Rounding Checklist Articles (n) (continued)

Significant Process of Care Outcomes	Positive/Improve	Negative/Worsen	Mixed
Endotracheal tube need assessment	1	-	-
Medication by mouth	1	-	-
Association between checklist responses and care delivered	1	-	-
Care coordination	1	-	-
Electrolytes	1	-	-
Goals of care	1	-	-
Fluid balance	1	-	-
Skin/wound care	1	-	-
Infection prevention	1	-	-
Need for devices	1	-	-
Central venous catheter dressing, cap, port needle change frequency	-	-	-
Daily oral care	1	-	-
Turning patient (pressure ulcer prevention)	1	-	-
Technical errors	-	-	-
Time to analgesia dosing	-	-	-
Faster ABGs after intubation	-	-	-
Concerns on rounds	1	-	-
Oxygenation index	1	-	-
Tracheostomy	1	-	-
Restraint order compliance	1	-	-
Hospital costs	1	-	-
Handover occurs at ICU bedside	-	-	-
Adequate handover preparation	-	-	-
Address patient's treatment preferences	-	-	-
Central venous catheter insertion bundle documentation	-	-	-
Head circumference measurement	-	1	-
**Total**	**84**	**7**	**0**

Table S7. Statistically Significant Process of Care Outcomes Reported by Transfer & Handover Checklist Articles (n)

Significant Process of Care Outcomes	Positive/Improve	Negative/Worsen	Mixed
Information transfer/sharing	10	-	-
Attendance	5	-	-
Sedation/sedation management	-	-	-
DVT/VTE prophylaxis	-	-	-
Low tidal volume ventilation/lung protective ventilation	-	-	-
Stress ulcer prophylaxis/ peptic ulcer prophylaxis	-	-	-
Omissions	4	-	-
Medication review/ reconciliation/ adjustments	1	-	-
Head of bed elevation	-	-	-
Central venous catheter removal/need assessment	-	-	-
Lab utilisation	-	-	-
Round/handover completion time	2	-	-
Spontaneous breathing trial	-	-	-
Pain/pain management	-	-	-
Glucose management	-	-	-
Antimicrobial/ antibiotic use	-	-	-
Antimicrobial/ antibiotic need assessment	-	-	-
Nutrition assessment	-	-	-
Weight	-	-	-
Urinary catheter removal/need assessment	-	-	-
Lab necessity/ diagnostics	-	-	-
Family updates/ communication	-	-	-
Occupational therapy/ physical therapy	-	-	-
Bowel protocol/ movement	-	-	-
Mechanical ventilation weaning, extubation planning/readiness	-	-	-

Table S7. Statistically Significant Process of Care Outcomes Reported by Transfer & Handover Checklist Articles (n) (continued)

**Significant Process of Care Outcomes**	**Positive/Improve**	**Negative/Worsen**	**Mixed**
Endotracheal tube need assessment	-	-	-
Medication by mouth	-	-	-
Association between checklist responses and care delivered	-	-	-
Care coordination	-	-	-
Electrolytes	-	-	-
Goals of care	-	-	-
Fluid balance	-	-	-
Skin/wound care	-	-	-
Infection prevention	-	-	-
Need for devices	-	-	-
Central venous catheter dressing, cap, port needle change frequency	-	-	-
Daily oral care	-	-	-
Turning patient (pressure ulcer prevention)	-	-	-
Technical errors	1	-	-
Time to analgesia dosing	1	-	-
Faster ABGs after intubation	-	-	-
Concerns on rounds	-	-	-
Oxygenation index	-	-	-
Tracheostomy	-	-	-
Restraint order compliance	-	-	-
Hospital costs	-	-	-
Handover occurs at ICU bedside	1	-	-
Adequate handover preparation	1	-	-
Address patient's treatment preferences	1	-	-
Central venous catheter insertion bundle documentation	-	-	-
Head circumference measurement	-	-	-
**Total**	**27**	**0**	**0**

Table S8. Statistically Significant Process of Care Outcomes Reported by CLABSI Prevention Checklist Articles (n)

Significant Process of Care Outcomes	Positive/Improve	Negative/Worsen	Mixed
Information transfer/sharing	-	-	-
Attendance	-	-	-
Sedation/sedation management	-	-	-
DVT/VTE prophylaxis	-	-	-
Low tidal volume ventilation/lung protective ventilation	-	-	-
Stress ulcer prophylaxis/ peptic ulcer prophylaxis	-	-	-
Omissions	-	-	-
Medication review/ reconciliation/ adjustments	-	-	-
Head of bed elevation	-	-	-
Central venous catheter removal/need assessment	1	-	-
Lab utilisation	-	-	-
Round/handover completion time	-	-	-
Spontaneous breathing trial	-	-	-
Pain/pain management	-	-	-
Glucose management	-	-	-
Antimicrobial/ antibiotic use	-	-	-
Antimicrobial/ antibiotic need assessment	-	-	-
Nutrition assessment	-	-	-
Weight	-	-	-
Urinary catheter removal/need assessment	-	-	-
Lab necessity/ diagnostics	-	-	-
Family updates/ communication	-	-	-
Occupational therapy/ physical therapy	-	-	-
Bowel protocol/ movement	-	-	-
Mechanical ventilation weaning, extubation planning/readiness	-	-	-

Table S8. Statistically Significant Process of Care Outcomes Reported by CLABSI Prevention Checklist Articles (n) (continued)

Significant Process of Care Outcomes	Positive/Improve	Negative/Worsen	Mixed
Endotracheal tube need assessment	-	-	-
Medication by mouth	-	-	-
Association between checklist responses and care delivered	-	-	-
Care coordination	-	-	-
Electrolytes	-	-	-
Goals of care	-	-	-
Fluid balance	-	-	-
Skin/wound care	-	-	-
Infection prevention	-	-	-
Need for devices	-	-	-
Central venous catheter dressing, cap, port needle change frequency	1	-	-
Daily oral care	-	-	-
Turning patient (pressure ulcer prevention)	-	-	-
Technical errors	-	-	-
Time to analgesia dosing	-	-	-
Faster ABGs after intubation	-	-	-
Concerns on rounds	-	-	-
Oxygenation index	-	-	-
Tracheostomy	-	-	-
Restraint order compliance	-	-	-
Hospital costs	-	-	-
Handover occurs at ICU bedside	-	-	-
Adequate handover preparation	-	-	-
Address patient's treatment preferences	-	-	-
Central venous catheter insertion bundle documentation	-	1	-
Head circumference measurement	-	-	-
**Total**	**2**	**1**	**0**

Table S9. Statistically Significant Process of Care Outcomes Reported by Airway Management Checklist Articles (n)

**Significant Process of Care Outcomes**	**Positive/Improve**	**Negative/Worsen**	**Mixed**
Information transfer/sharing	-	-	-
Attendance	-	-	-
Sedation/sedation management	-	-	-
DVT/VTE prophylaxis	-	-	-
Low tidal volume ventilation/lung protective ventilation	-	-	-
Stress ulcer prophylaxis/ peptic ulcer prophylaxis	-	-	-
Omissions	-	-	-
Medication review/ reconciliation/ adjustments	-	-	-
Head of bed elevation	-	-	-
Central venous catheter removal/need assessment	-	-	-
Lab utilisation	-	-	-
Round/handover completion time	-	-	-
Spontaneous breathing trial	-	-	-
Pain/pain management	-	-	-
Glucose management	-	-	-
Antimicrobial/ antibiotic use	-	-	-
Antimicrobial/ antibiotic need assessment	-	-	-
Nutrition assessment	-	-	-
Weight	-	-	-
Urinary catheter removal/need assessment	-	-	-
Lab necessity/ diagnostics	-	-	-
Family updates/ communication	-	-	-
Occupational therapy/ physical therapy	-	-	-
Bowel protocol/ movement	-	-	-
Mechanical ventilation weaning, extubation planning/readiness	-	-	-

Table S9. Statistically Significant Process of Care Outcomes Reported by Airway Management Checklist Articles (n) (continued)

**Significant Process of Care Outcomes**	**Positive/Improve**	**Negative/Worsen**	**Mixed**
Endotracheal tube need assessment	-	-	-
Medication by mouth	-	-	-
Association between checklist responses and care delivered	-	-	-
Care coordination	-	-	-
Electrolytes	-	-	-
Goals of care	-	-	-
Fluid balance	-	-	-
Skin/wound care	-	-	-
Infection prevention	-	-	-
Need for devices	-	-	-
Central venous catheter dressing, cap, port needle change frequency	-	-	-
Daily oral care	-	-	-
Turning patient (pressure ulcer prevention)	-	-	-
Technical errors	-	-	-
Time to analgesia dosing	-	-	-
Faster ABGs after intubation	1	-	-
Concerns on rounds	-	-	-
Oxygenation index	-	-	-
Tracheostomy	-	-	-
Restraint order compliance	-	-	-
Hospital costs	-	-	-
Handover occurs at ICU bedside	-	-	-
Adequate handover preparation	-	-	-
Address patient's treatment preferences	-	-	-
Central venous catheter insertion bundle documentation	-	-	-
Head circumference measurement	-	-	-
**Total**	**1**	**0**	**0**

Table S10. Articles by Type of Study

Checklist Type	Implementation	Validation	Other[a]
Rounding, n (%)	45 (86.5)	2 (3.8)	5 (9.6)
Delirium Screening, n (%)	1 (3.1)	23 (71.9)	8 (25.0)
Transfer & Handover, n (%)	19 (95.0)	-	1 (5.0)
CLABSI Prevention, n (%)	8 (72.7)	-	3 (27.3)
Airway Management, n (%)	5 (62.5)	-	3 (37.5)

[a]The "other" category contains the remaining studies that were not implementation or validation studies. These studies were predominantly systematic reviews and meta-analyses and surveys.

Table S11. Checklist Development Method

Checklist Type	No iterative development or not specified	Checklist from previous study	Checklist already in place	Iterative development
Rounding, n (%)	15 (31.9)	10 (21.3)	11 (23.4)	11 (23.4)
Delirium Screening, n (%)	-	24 (100)	-	-
Transfer & Handover, n (%)	7 (36.8)	5 (26.3)	-	7 (36.8)
CLABSI Prevention, n (%)	6 (75.0)	2 (25.0)	-	-
Airway Management, n (%)	1 (20.0)	1 (20.0)	-	3 (60.0)

Definitions

- *No iterative development or not specified:* checklists that do not fit into one of the following three categories.
- *Checklists derived from a previous study:* checklists that have been sourced directly from literature and may have undergone minor modifications before implementation.
- *Checklist already in place:* checklists that already existed at the institution and may have undergone a slight update before implementation.
- *Iterative development:* a cyclical development process in which a checklist underwent multiple rounds of revisions based on feedback from testing, expert feedback, or any other source of information that informs the revisions.

Table S12. Concurrent Interventions

Checklist Type	None	Education/ Training	Feedback on Performance or Outcomes	Other
Rounding, n (%)	16 (34.0)	24 (51.1)	9 (19.1)	19 (40.4)
Delirium Screening, n (%)	6 (25.0)	18 (75.0)	-	-
Transfer & Handover, n (%)	5 (26.3)	10 (52.6)	-	13 (68.4)
CLABSI Prevention, n (%)	1 (12.5)	6 (75.0)	4 (50.0)	5 (62.5)
Airway Management, n (%)	2 (40.0)	3 (60.0)	1 (20.0)	2 (40.0)

Figure S1. Articles by Region (n)

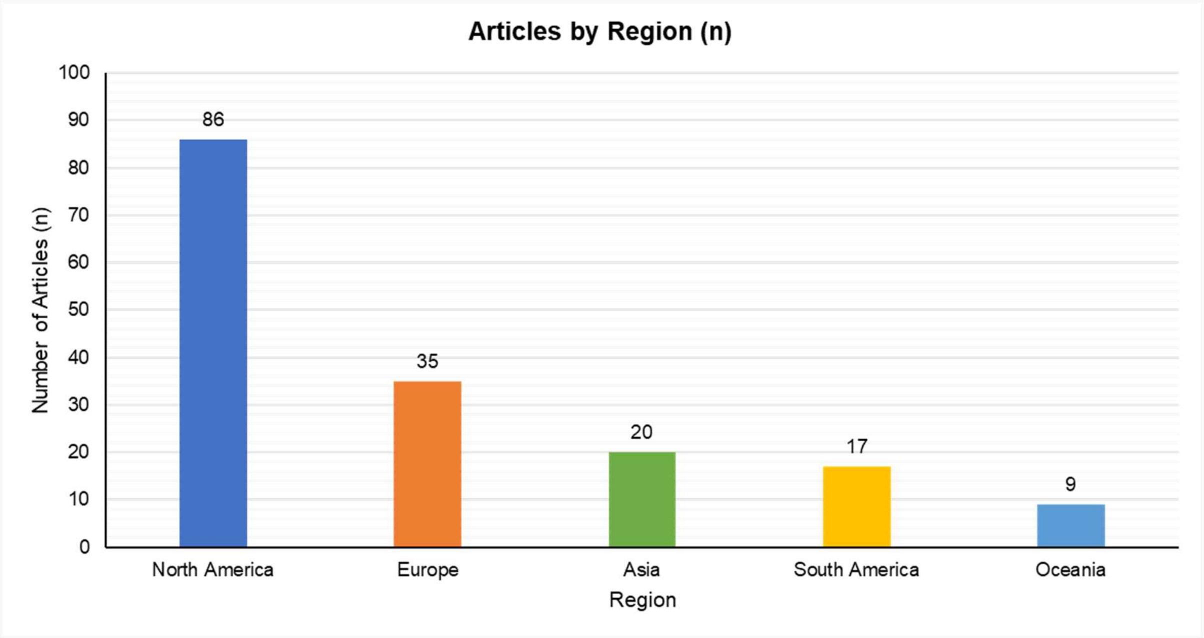

Figure S2. Articles by Year (n)

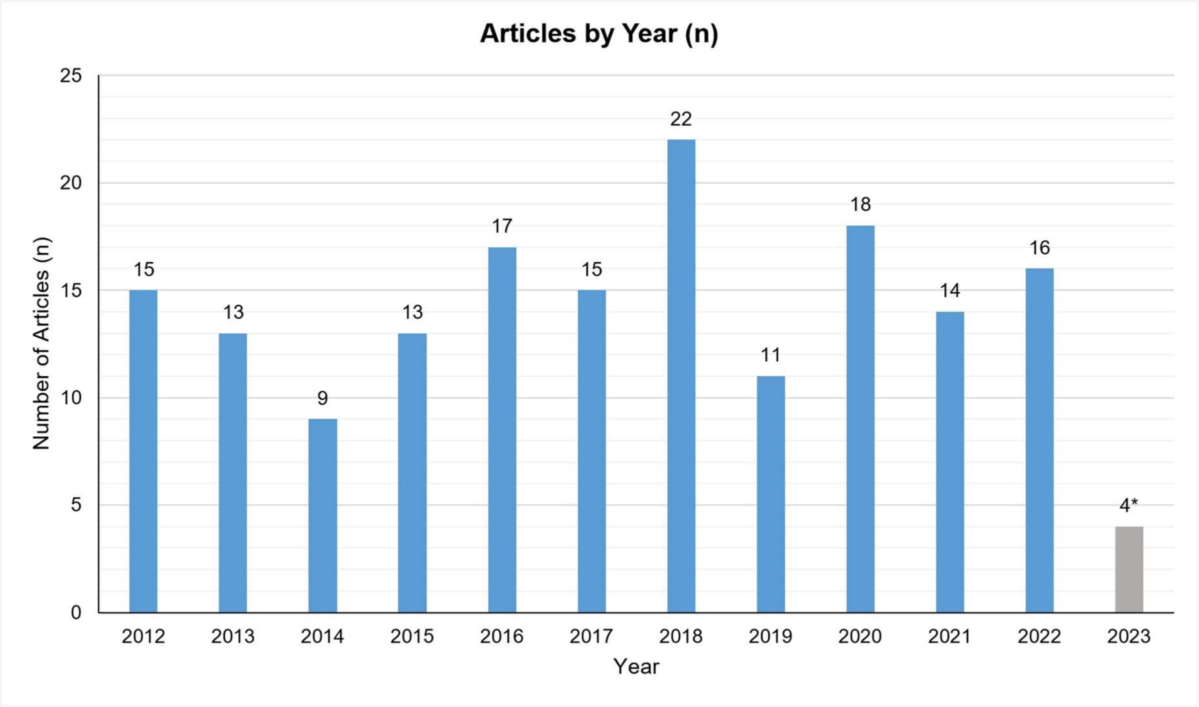

Figure S3. Length of Checklists

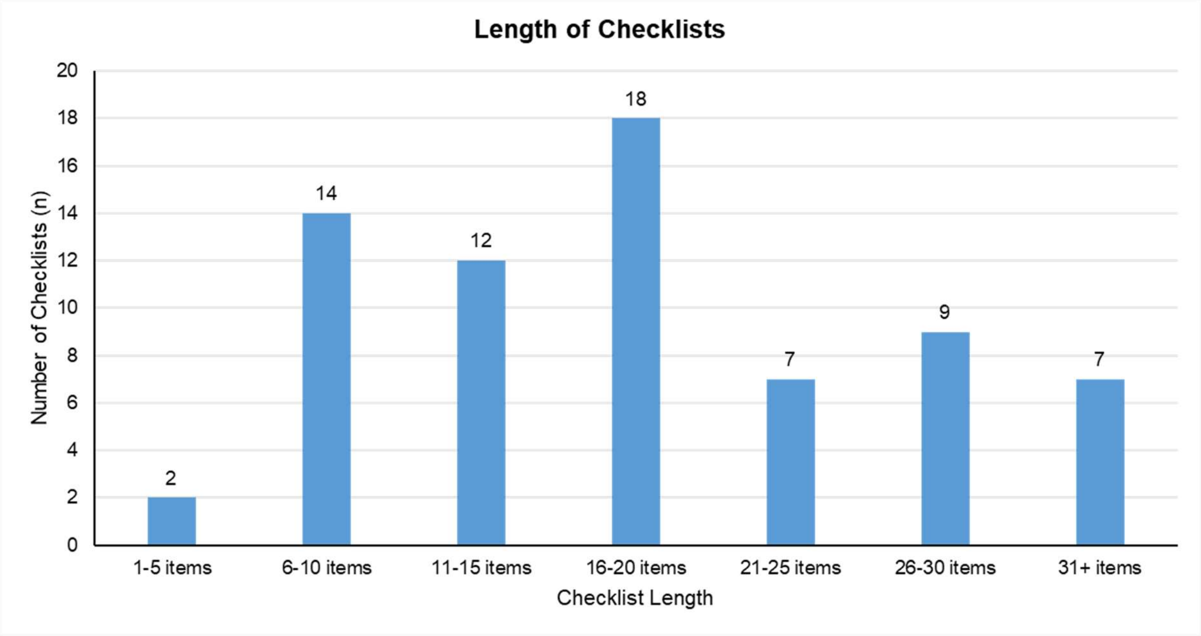

Figure S4. Articles Using Electronic Checklists

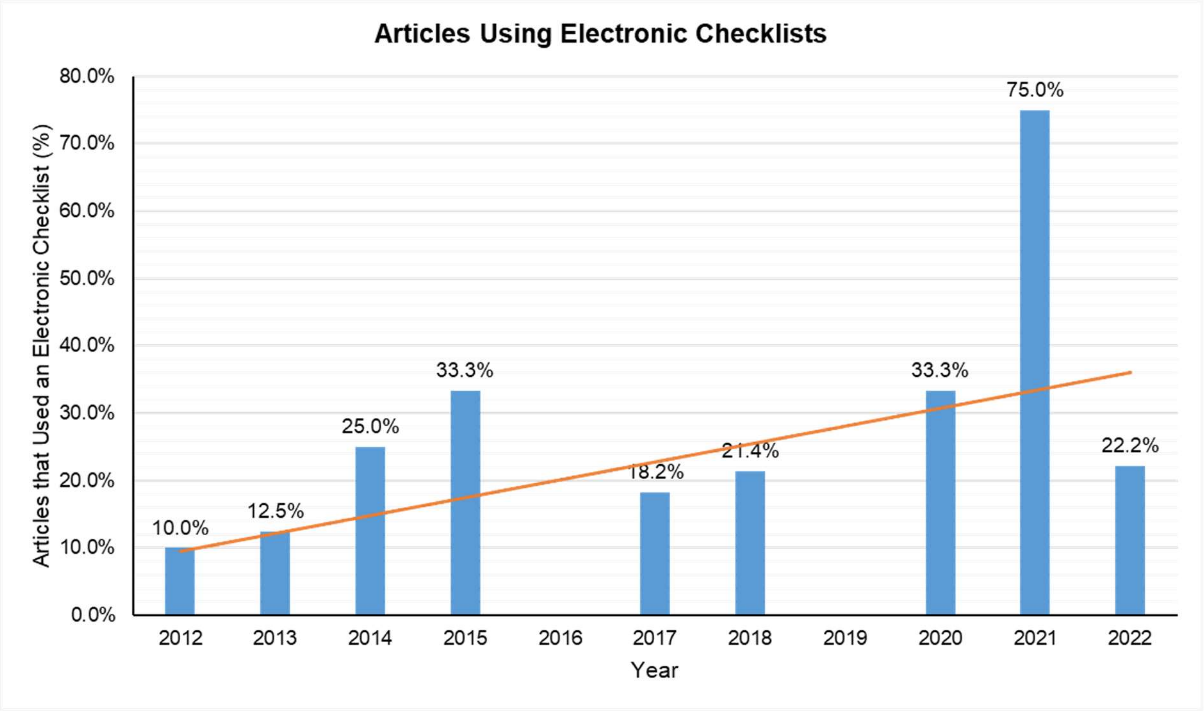

Figure S5. Articles Using Physical Checklists

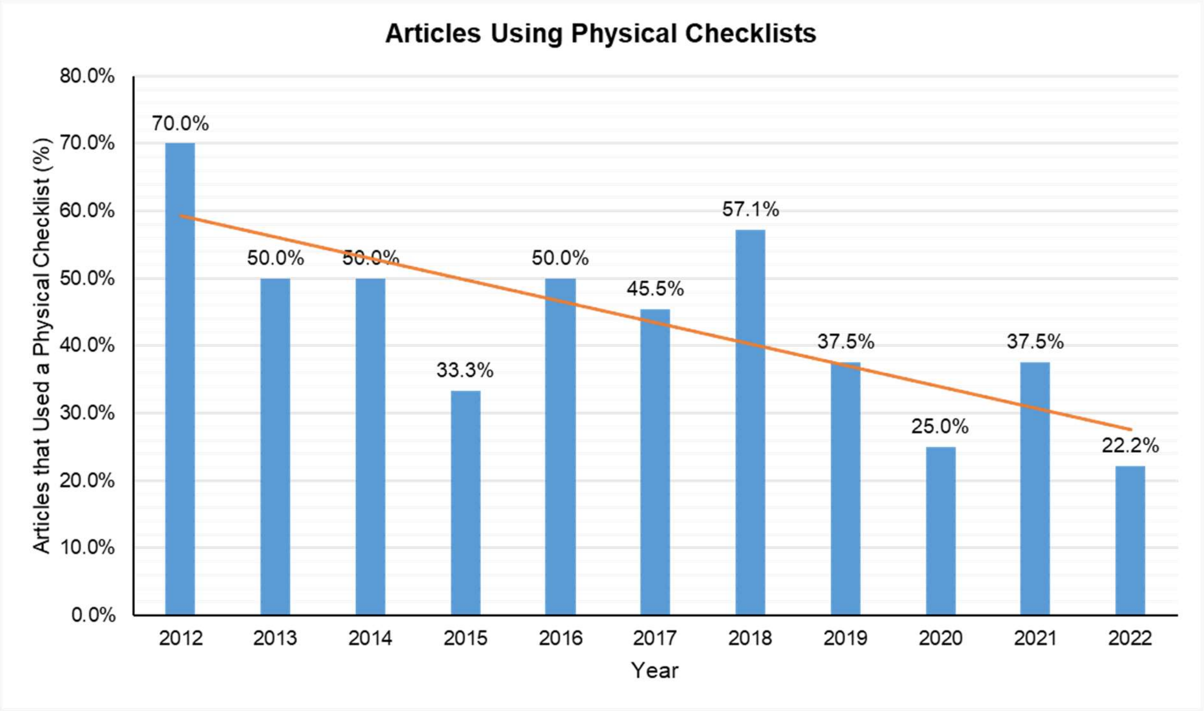

Supplement: Supplementary file 1 — Additional file 1. Online supporting information. [file 13054_2023_4758_MOESM1_ESM.pdf]
